# Crystal structure thermal evolution and novel orthorhombic phase of methylammonium lead bromide, CH_3_NH_3_PbBr_3_

**DOI:** 10.1038/s41598-022-21544-2

**Published:** 2022-11-04

**Authors:** Carmen Abia, Carlos A. López, Laura Cañadillas-Delgado, María T. Fernández-Diaz, José A. Alonso

**Affiliations:** 1 Instituto de Ciencia de Materiales de Madrid, CSIC, Cantoblanco, 28049 Madrid, Spain; 2 INTEQUI, (UNSL-CONICET) and Facultad de Química, Bioquímica y Farmacia, UNSL, Almirante Brown 1455, 5700 San Luis, Argentina; 3 Institut Laue Langevin, 38042 Grenoble Cedex, France

**Keywords:** Chemistry, Energy science and technology, Materials science

## Abstract

Methylammonium (MA) lead trihalide perovskites, CH_3_NH_3_PbX_3_ (X = I, Br, Cl), have emerged as a new class of light-absorbing materials for photovoltaic applications, reaching efficiencies of 23% when implemented in solar cell heterojunctions. In particular, MAPbBr_3_ is a promising member with a large bandgap that gives rise to a high open circuit voltage. Here we present a structural study from neutron diffraction (ND) data of an undeuterated MAPbBr_3_ specimen, carried out to follow its crystallographic behaviour in the 2–298 K temperature range. Besides the known crystallographic phases, i.e. the high-temperature *Pm*$\overline{3}$*m* cubic structure, the intermediate *I*4*/mcm* tetragonal symmetry and the low-temperature *Pnma* orthorhombic phase, we additionally identified, from a detailed sequential ND analysis, a novel intermediate phase within the 148.5–154.0 K temperature range as an orthorhombic *Imma* structure, early associated with a coexistence of phases. Moreover, our ND data allowed us to unveil the configuration of the organic MA units and their complete localization within the mentioned temperature range, thus improving the crystallographic description of this compound. The evolution with temperature of the H-bonds between the organic molecule and the inorganic cage is also followed. A deep knowledge of the crystal structure and, in particular, the MA conformation inside the perovskite cage seems essential to establish structure–property correlations that may drive further improvements.

## Introduction

The recently discovered organic–inorganic hybrid perovskites are promising materials for the next generation of solar cells. Amongst their advantages, it is remarkable their affordable manufacture and their good performance^1–5^. The general formula for hybrid perovskites is ABX_3_, in which B (metals) and X (halogens) constitute BX_6_ octahedra, building a three-dimensional framework that encloses the organic cation A. Given the wide choice of components, these hybrid perovskite materials present a great chemical flexibility.

The use of perovskite-based photovoltaic materials in solar cell devices began with Miyasaka et al. introduction of MAPbX_3_ (MA: CH_3_NH_3_^+^ and X = Br, I) as a sensitizer in an electrolyte-based solar cell structure^2^. However, due to the corrosion of the perovskites by the liquid electrolyte, the power conversion efficiency (PCE) and cell stability were low. To overcome this issue, the liquid electrolyte was replaced with a solid hole-transporting material^2^, which resulted in a better PCE and enhanced stability. Subsequently, great efforts have gone into improving these halide perovskite-based solar cells, with a variety of cell designs being produced in recent years, boosting the PCEs to 23%^6^. Methylammonium (MA: CH_3_NH_3_^+^) lead triiodide, CH_3_NH_3_PbI_3_ (known as MAPbI_3_ or MAPI), is the most investigated hybrid perovskite and the best option for solar applications to date. It has a suitable bandgap of ~ 1.6 eV, which enables a broad absorption range over the entire visible light spectrum^7^. Unfortunately, it exhibits a fast degradation when exposed to UV radiation at mild temperatures and has a low moisture tolerance, which constitutes an important drawback for its commercialization^8–11^. Alternative options in the chemical composition of the perovskites have been proposed in order to overcome this problem. With a broad band gap of 2.2 eV and a high open-circuit voltage (Voc ≈ 1.2–1.5 V), CH_3_NH_3_PbBr_3_ is a viable alternative to CH_3_NH_3_PbI_3_. It also has a long exciton diffusion length (> 1.2 μm), allowing an efficient charge transport in devices^12^. In addition, compared to the pseudocubic CH_3_NH_3_PbI_3_, CH_3_NH_3_PbBr_3_ demonstrates better stability towards air and moisture, due to its stable cubic phase at room temperature and low ionic mobility, in which inherent lattice strain provides an avenue for increased diffusion^12–15^. These properties may compensate for a relatively high exciton binding energy (76 meV) and reduced light absorption beyond its band edge at 550 nm, explaining why CH_3_NH_3_PbBr_3_ solar cells have a more limited efficiency^13,14,16–18^.

Because the perovskites' crystallochemistry is so closely linked to their macroscopic phenomenology, it is critical to understand the crystallographic structure and the many phase transitions they might undergo through as a function of temperature. It is well known the importance to unveil the details of the crystal structure, such as MA delocalisation, tilting of polyhedra, phase transitions, etc., in relation to the physicochemical behaviour and macroscopic phenomenology. As other perovskites, MAPbBr_3_ presents three known phases: a high temperature cubic phase; an intermediate tetragonal phase and a low temperature orthorhombic phase. The first structural studies of MAPbBr_3_ that were made using diffraction techniques were single crystal x-ray experiments or neutron experiments on deuterated samples^5,19,20^. Recently, we followed the phase transitions by synchrotron x-ray diffraction, and a complete elucidation of MA conformation was presented from neutron powder diffraction in an undeuterated sample at room temperature^21^. That work confirmed the three mentioned phases, orthorhombic (*Pnma*), tetragonal (*I*4/*mcm*) and cubic (*Pm*$\overline{3}$*m*); however a transient phase was observed at 150 K, which could not be resolved^21^. Afterward, Yang et al*.* reported the crystal structure from single crystal neutron diffraction in the 95–300 K range^22^. However, up to now the MA position and its interaction with PbBr_6_ inorganic lattice is unknown in the low-temperature orthorhombic polymorph. In this symmetry, the MA unit is localized in a unique position, in contrast with the delocalization described in the tetragonal and cubic phases, and the organic–inorganic interactions at this symmetry are known only from theoretical studies.

Moreover, for this compound, an additional crystallographic configuration has been reported in the literature within the 148–155 K temperature region, which remains under debate. Older reports informed an intermediate tetragonal phase (*P*4/*mmm*) between the orthorhombic and tetragonal (*I*4/*mcm*) from single crystal x-ray diffraction and calorimetric analysis^23–25^. Then, Wang et al*.*^26^ reported on the coexistence of the two different crystallographic phases, tetragonal and orthorhombic, around that range by using x-ray diffraction measurements and temperature-dependent absorption/PL spectroscopy. Yang et al*.*^22^ also suggested the coexistence of the tetragonal and orthorhombic phases, after detecting a fluctuation of the (220) tetragonal peak between ≈ 140 and 155 K, by performing neutron diffraction measurements on a MAPbBr_3_ single crystal. Similarly, Gou et al*.*^27^ also proposed a tetragonal intermediate phase from Raman spectroscopy and single crystal XRD data, suggesting an incommensurate phase. In a more recent study, Wiedemann et al*.*^28^ described the intermediate structure (at 150 K) as an incommensurate modulated structure within the (3 + 1)D superspace group *Imma*(00γ)s00, deduced from single-crystal x-ray diffraction.

In this work, we present the results of the sequential neutron powder diffraction (NPD) data collection in a powdered and non-deuterated MAPbBr_3_ sample in the 2–250 K temperature range. An additional measurement was carried out at 300 K using a single crystal. On the one hand, we deepen on the knowledge on the already described cubic *Pm*$\overline{3}$*m*, tetragonal *I4/mcm* and orthorhombic *Pnma* phases. Moreover, from additional sequential ND measurements with higher resolution in temperature, we resolved the intermediate structure as a commensurate orthorhombic *Imma* phase. This model accurately describes the crystallographic structure in the controversial 148–155 K temperature range, completely elucidating the configuration of the MA units.

## Experimental

The crystal growth of MAPbBr_3_ was carried out by the inverse temperature crystallization method^29^, as reported in our previous publication^21^. Stoichiometric amounts of PbBr_2_ and MABr reacted in a 1 M solution using dimethylformamide (DMF) as a solvent, obtaining orange single crystals. These crystals were ground to yield a microcrystalline powder, suitable for the powder diffraction experiments. Some crystals were kept for a single-crystal study.

The neutron diffraction (ND) experiments were made in the Institut Laue Langevin (ILL), Grenoble, France. The thermal evolution of the crystallographic structure was studied with neutron powder diffraction (NPD); sequential NPD patterns from 2 to 250 K with a temperature interval of ≈ 4.5 K were measured on the high-flux D20 diffractometer with a wavelength 1.540 Å and a take-off angle of 90°. An additional sequential analysis around critical phase transition was measured from 145 to 157 K with a temperature interval of ≈ 0.5 K. About 2 g of non-deuterated sample was contained in a 6 mm diameter vanadium can. The coherent scattering lengths for Pb, Br, C, N and H were, 9.405, 6.795, 6.646, 9.36 and − 3.739 fm, respectively. NPD diffraction patterns were analysed with the Rietveld method using the *FullProf* program^30,31^. In order to generate the shape of the diffraction peaks of the patterns collected, a pseudo-Voigt function was selected. The background was interpolated from areas with no reflections. The parameters simultaneously refined were: scale factor, background coefficients, zero-point error, pseudo-Voigt corrected for asymmetry parameters, positional coordinates, anisotropic displacement factors and occupancy factors. The high contrast of H scattering lengths allowed the full elucidation of the organic MA cations.

Moreover, an additional data collection at room temperature (RT) was carried out in the single-crystal neutron diffractometer D19 (ILL, Grenoble), with a wavelength of 0.96 Å. For this experiment, a suitable ‘‘neutron-sized’’ crystal with dimensions of 1 × 1 × 1 mm^3^, approximately, was mounted on a pin, and the measurements were carried out with a neutron wavelength of 0.96 Å. The strong peaks were found with the ILL program PFIND, and indexed with the DIRAX^32^ program; the orientation matrix, the unit-cell parameters and the χ, ω and detector offsets were post-refined with the ILL program RAFD19^33,34^. After that, a sequence of approximately 801 ω-scans at a number of positions of ϕ and χ were collected to obtain high data completeness. The reflections were integrated using the ILL program RETREAT. The crystal attenuation correction was done with the D19FACE, D19ABS and D19ABSCAN programs, after manually indexing the crystal faces and checking them with PLATON software^35^.

## Results and discussion

### Calorimetric measurements (DSC)

**Figure 1:**
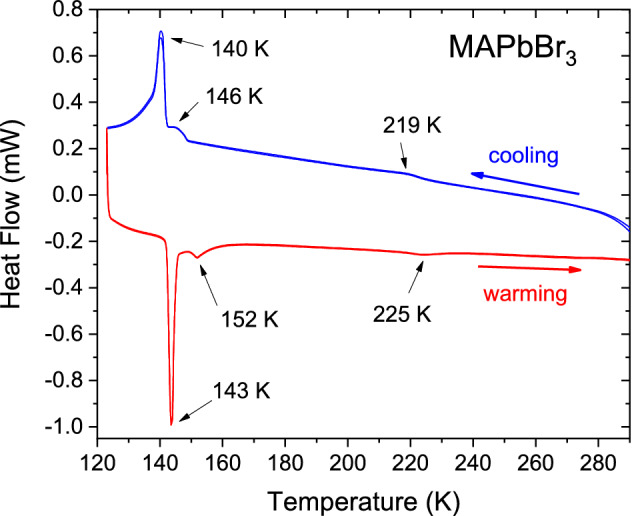
Warming and cooling cycles of the DSC curves of MAPbBr_3_, showing the reversible phase transitions.

The calorimetric measurements were performed to confirm the expected phase transitions reported in MAPbBr_3_. Figure 1 shows the DSC curves below RT, corresponding to two consecutive cooling–warming cycles. There are three thermal events, at 219, 146 and 140 K in the cooling run, and 143, 152 and 225 K in the warming runs, with low hysteresis. The observed temperatures are close to those reported by Onoda-Yamamuro et al*.* from heat capacity^24^. Besides, these temperature values are in agreement with previous structural results as it was discussed in the Introduction Sect. ^21–25^. Of the three events only one is sharp and intense, hence, it can be associated with a high caloric transition. This fact allows supposing that this transition involves strong changes in H-bond interactions, which can be related to the order–disorder changes in MA cation.

### Structural analysis at room temperature (single crystal ND)

The room temperature crystal structure analysis carried out from single-crystal ND data confirms the cubic *Pm*$\overline{3}$*m* space group. The three possibilities of MA delocalization, i.e. [100], [110] and [111] were tested, and the best fit was achieved when the MA unit is aligned along [110] direction. This fact is in agreement with our previous results from neutron powder refinements^21^. Furthermore, the single-crystal data allow us to refine the C–N displacement in the inorganic lattice in addition to the anisotropic displacement factors. The crystallographic results and the main Br···H distances and angles are listed in Tables 1 and 2, respectively.

**Table 1:** Crystallographic data for MAPbBr_3_ phase in cubic system (*Pm*$\overline{3}$*m*) from single crystal ND at 298 K.

	*x*	*y*	*z*	*U*_*eq*_	*f*_*occ*_	
Pb	0	0	0	0.0306 (2)	1	
Br	0.5	0	0	0.1027 (3)	1	
N1	0	0.6084 (3)	0.6084(3)	0.0667 (5)	0.0833	
C2	0	0.4416 (2)	0.4416 (2)	0.0667 (5)	0.0833	
H11	0.5	0.557 (3)	0.739 (2)	0.13 (3)	0.0417	
H12	0.648 (1)	0.711 (1)	0.584 (2)	0.11 (2)	0.0417	
H21	0.5	0.527 (3)	0.278 (2)	0.13 (3)	0.0417	
H22	0.352 (1)	0.335 (2)	0.454 (3)	0.11 (2)	0.0417	
w_RF_^2^ = 5.83%, χ^2^ = 1.7, R_Bragg_ = 4.04%						

	*U*^*11*^	*U*^*22*^	*U*^*33*^	*U*^*12*^	*U*^*13*^	*U*^*23*^
Atomic displacement parameters (Å^2^)						
Pb	0.0306 (2)	0.0306 (2)	0.0306 (2)	0	0	0
Br	0.0200 (2)	0.1440 (4)	0.1440 (4)	0	0	0
N1	0.0548 (4)	0.0726 (5)	0.0726 (5)	0	0	− 0.0548 (5)
C2	0.0548 (4)	0.0726 (5)	0.0726 (5)	0	0	− 0.0548(5)
H11	0.27 (6)	0.03 (3)	0.10 (1)	0	0	− 0.03 (3)
H12	0.103 (9)	0.103 (9)	0.13 (3)	− 0.05 (1)	− 0.03 (2)	0.005 (2)
H21	0.27 (6)	0.03 (3)	0.10 (1)	0	0	− 0.03 (3)
H22	0.103 (9)	0.103 (9)	0.13 (3)	− 0.05 (1)	− 0.03 (2)	0.005 (2)

*a* = 5.9259(1) Å, and V = 208.10(1) Å^3^.

**Table 2:** Possible H-bond distances of MA at 300 K.

	Label	Distance (Å)	Angle (°)
**–NH**_**3**_			
NH11···Br1	1	3.05 (1)	99.0 (7)
NH11···Br1	2	3.36 (1)	117.7 (1)
NH12···Br1	3	2.74 (1)	174.8 (4)
**–CH**_**3**_			
CH21···Br1	4	3.53 (1)	90.2 (5)
CH21···Br1	5	3.39 (1)	114.1 (3)
CH22···Br1	6	2.89 (1)	172.0 (4)

**Figure 2:**
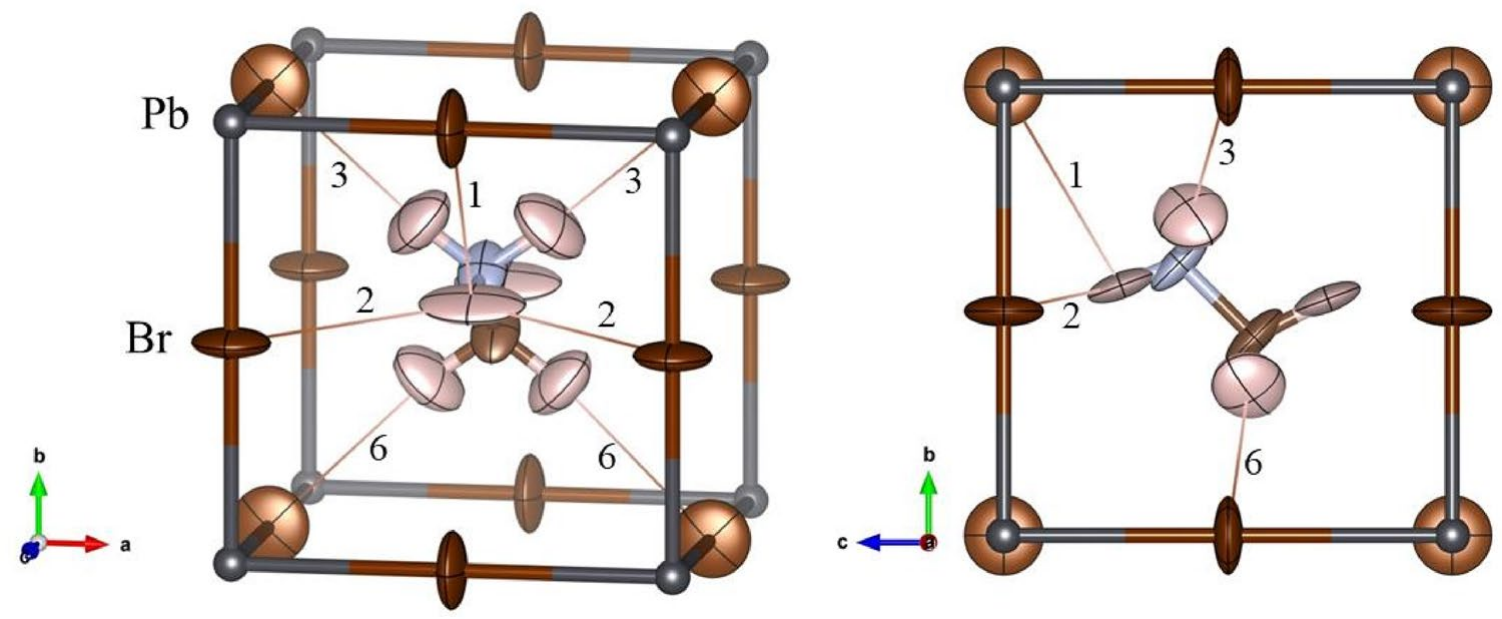
Two views of the cubic crystal structure of MAPbBr_3_, highlighting the H-bond interactions with adjacent Br atoms approximately along [101] (*left*) and [010] (*right*) directions. The numbers correspond to the labels indicated in Table 2.

Theoretical studies of MAPbBr_3_ differ regarding the MA alignment in cubic symmetry. Varadwaj et al*.*^36^ found that the [100] and [111] orientations are the most energetically favourable. However, Yin et al*.*^37^ reported that the MA is aligned along [110] from ab initio calculations and Raman spectroscopy. They also observed that MA only interacts with the inorganic lattice by H-bonds through –NH_3_, and these distances are in agreement with our results. However, the values in Table 2 reveal an additional H-bond through –CH_3_ by H22 atoms. Figure 2 illustrates a unique MA in the PbBr_3_ cage where the deduced H-bond interactions are highlighted and labelled with the numbers listed in Table 2. On the other hand, additional information on the MA behaviour can be obtained from the anisotropic atomic displacement factors. The H12 is slightly flattened in the N–H12···Br direction (label 6); in contrast, H11 is slightly stretched in the H-bond labelled as 1, while it is very stretched in the H-bond labelled 2 (see Table 2 and Fig. 2). This last behaviour is in agreement with the hardness of H-bond interaction, as can be deduced from the distance and angles. Finally, the anisotropic displacement of N and C atoms suggests that the MA unit could rotate in the [100] direction; this movement implies that the MA units move over the [100] configurations. This fact suggests an energetic similarity between [110] and [100] positions in this phase. Besides DFT evidences, this behaviour was also observed by us from synchrotron X-ray diffraction, where the MA evolved from [110] to [100] directions below room temperature^21^.

### Structural analysis at 2 K

**Figure 3:**
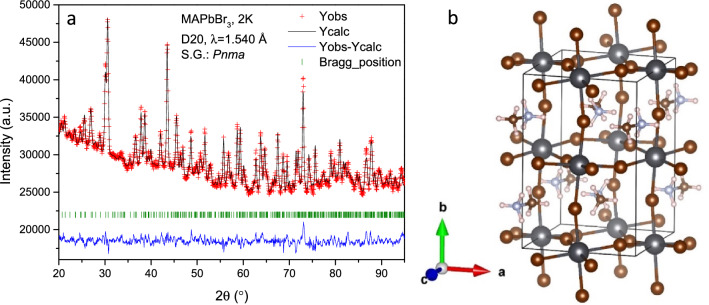
(**a**) Observed (crosses) calculated (black line) and difference (blue line) profiles after the Rietveld refinement from NPD data at 2 K, corresponding to orthorhombic (*Pnma*) symmetry. (**b**) View of the orthorhombic unit-cell of the corresponding crystallographic model.

The structural investigation performed at very low temperature, where the methylammonium’s mobility is minimized, allows making a detailed analysis of H-bond interactions between the MA and the PbBr_6_ inorganic framework. For this purpose, a high statistics NPD pattern was collected at 2 K at D20 powder diffractometer. Taking into account the previous results at low temperatures, MAPbBr_3_ should be at the orthorhombic symmetry in the *Pnma* space group^19,21^. The initial Le Bail refinements confirmed this symmetry; therefore, according to this model, the lead atoms are allocated in 4*b* (0,1/2,0) Wyckoff site and the bromides in 4*c* (*x*,1/4,*z*) and 8*d* (*x*,*y*,*z*) sites. This space group presents anti-phase octahedral tilts along *a* and *c*-axis and an in-phase octahedral tilt along *b*-axis, typified as a^–^b^+^a^–^ in the Glazer’s notation^38^. A subsequent analysis, taking into account this inorganic framework, is used to obtain the missing nuclear density (scattering length density) in the lattice from Difference Fourier Maps. Figure S1 (supplementary information) illustrates the positive (yellow) and negative (light blue) isosurfaces corresponding to the carbon/nitrogen and hydrogen atoms, respectively. These densities match the methylammonium cation and they allow to locate the atoms: C and N are at 4*c* (*x*,1/4,*z*) and H atoms distributed in 4*c* (*x*,1/4,*z*) and 8*d* (*x*,*y*,*z*) sites. The NPD pattern was successfully fitted with this model, as illustrated in Fig. 3. The main crystallographic data are shown in Table 3. The structure is similar to the one reported by Swainson et al*.*^19^ in a deuterated sample at 11 K and by Yang et al*.*^22^ at 95 K. It is important to remark that, in contrast with previous reports, no constraints were used in the refinements of the atomic position of the MA group, e.g. with no rigid body considerations.

**Table 3:** Crystallographic data for MAPbBr_3_ phase in orthorhombic system (*Pnma*) from NPD at 2 K.

	*x*	*y*	*Z*	*U*_*iso*_	*f*_*occ*_
Pb1	0	0	0.5	0.002 (1)	1
Br1	0.9689 (8)	0.25	0.4839 (8)	0.002 (1)	1
Br2	0.2981 (5)	0.0277 (3)	0.7106 (5)	0.002 (1)	1
N1	0.5500 (7)	0.25	0.5971 (7)	0.005 (1)	1
H11	0.682 (2)	0.25	0.596 (2)	0.032 (2)	1
H12	0.512 (2)	0.1795 (5)	0.652 (1)	0.032 (2)	1
C2	0.481 (1)	0.25	0.4336 (8)	0.005 (1)	1
H21	0.348 (2)	0.25	0.431 (2)	0.032 (1)	1
H22	0.523 (2)	0.1795 (5)	0.378 (1)	0.032 (1)	1
R_p_ = 0.83%, R_wp_ = 1.09%, χ^2^ = 3.64, R_Bragg_ = 6.81%					

*a* = 7.9335(4) Å, *b* = 11.8301(5) Å, *c* = 8.5743(3) Å and V = 804.74(5) Å^3^.

**Figure 4:**
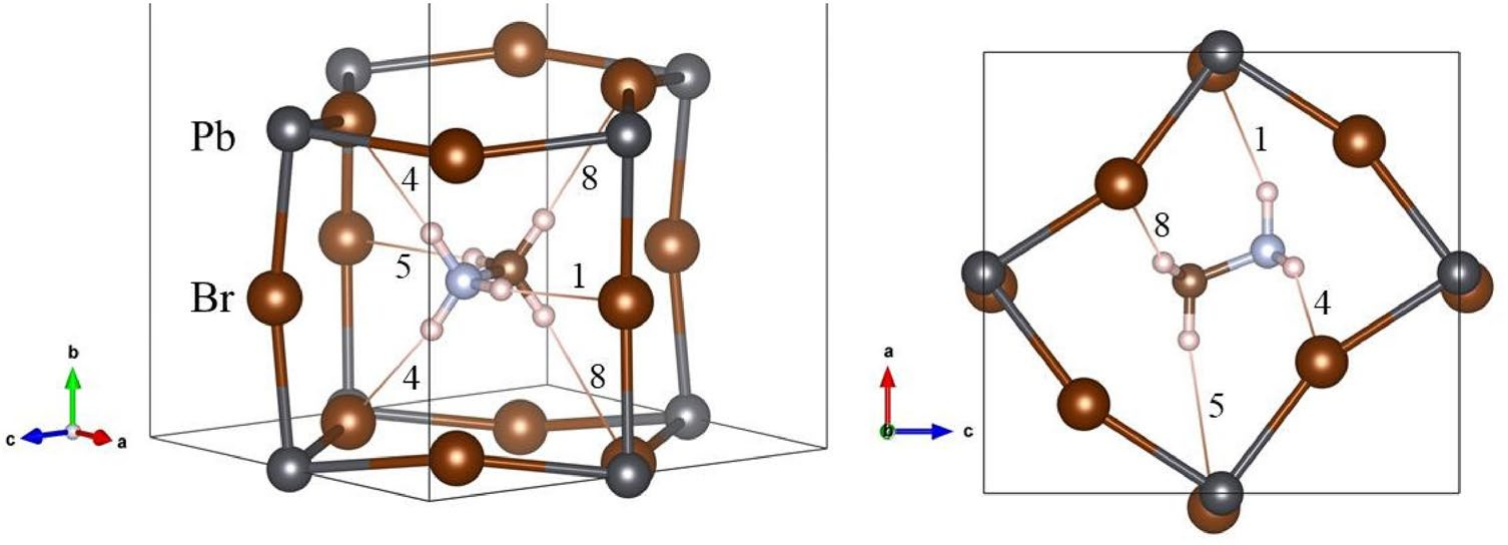
Two views of the orthorhombic crystal structure of MAPbBr_3_, highlighting the H-bond interactions with adjacent Br atoms approximately along [101] (*left*) and [010] (*right*) directions. The numbers correspond to the labels indicated in Table 2.

The H···Br distances and Br···H–N (and C) angles are listed in Table 4. From these distances and angles, it is possible to deduce that the H-bonds exist only in the H11···Br1 and H12···Br2 atom pairs for –NH_3_ and in H21···Br1 and H22···Br2 for –CH_3_ group, labelled as 1, 4, 5 y 8 respectively. Figure 4 illustrates the MA unit in the PbBr_3_ perovskite cage, where these four H-bonds are highlighted and numbered. These distances show that the MA unit is shifted, such that the (N)H···X distances are shorter than (C)H···X ones, which is expected considering the greater electronegativity of N respect to C. This displacement is 0.17 Å, considering the difference between the centres of MA and the PbBr_3_ perovskite cage. Additionally, these distances can be compared with those obtained by from DFT calculations. Varadwaj et al*.*^36^ found theoretically the same four H-bonds interactions with the following values: 2.501 Å and 2.446 Å for (N)H···Br and 3.005 Å and 2.916 Å for (C)H···Br. These distances and the angles reported from DFT are in agreement with those experimentally obtained in the present work.

**Table 4:** Possible H-bond distances of MA at 2 K.

	Label	Distance (Å)	Angle (°)
**–NH**_**3**_			
NH11···Br1	1	2.47 (2)	157.6 (7)
NH11···Br2	2	3.24 (1)	106.2 (4)
NH12···Br1	3	3.25 (1)	105.5 (4)
NH12···Br2	4	2.52 (1)	152.4 (6)
**–CH**_**3**_			
CH21···Br1	5	3.04 (2)	170.1 (7)
CH21···Br2	6	3.96 (1)	107.5 (3)
CH22···Br1	7	3.24 (1)	101.3 (5)
CH22···Br2	8	2.93 (9)	164.8 (6)

### Sequential analysis from 2 to 250 K (ΔT ≈ 4.5 K)

**Figure 5:**
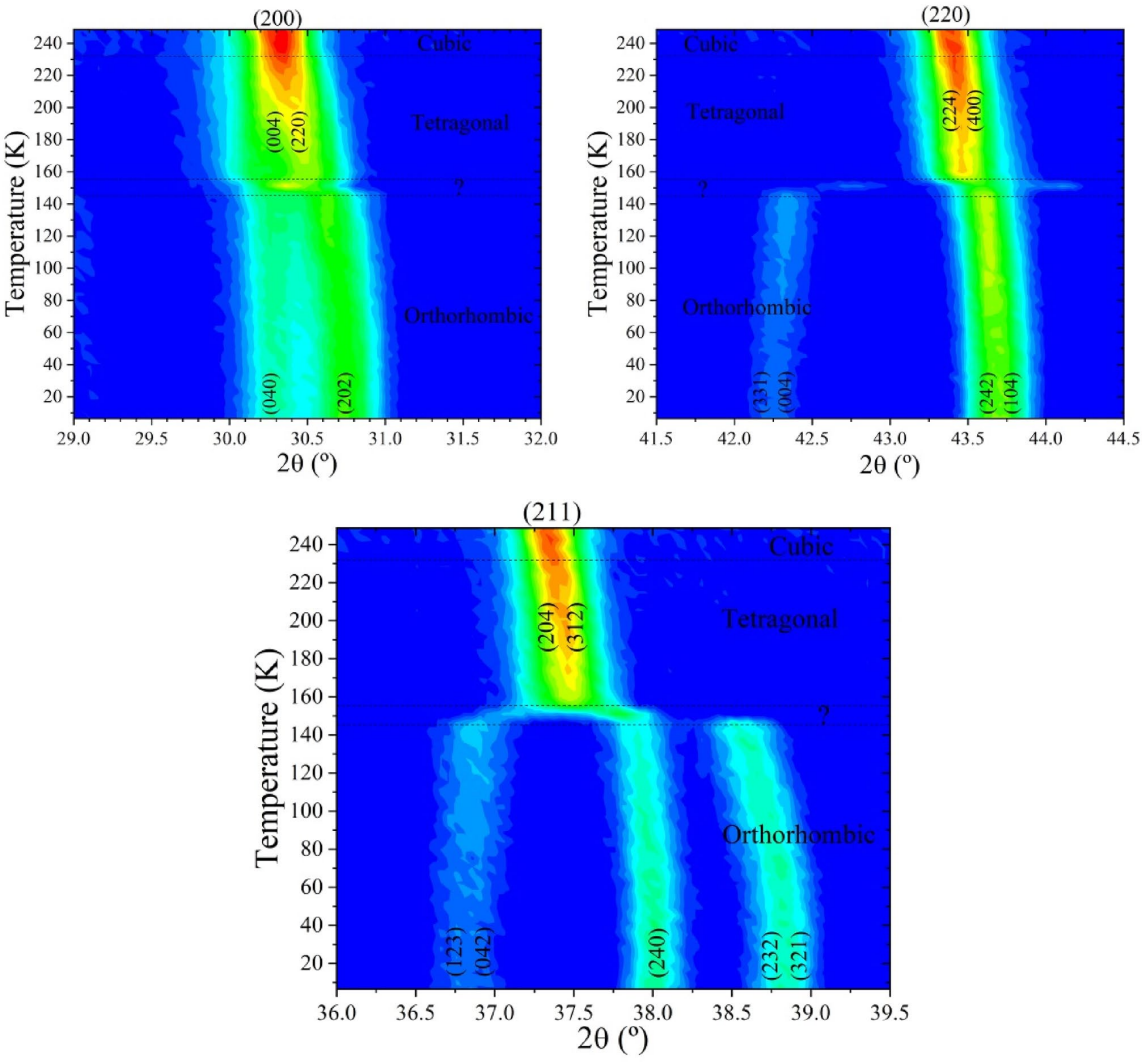
Thermal evolution of neutron diffraction patterns from 2 to 250 K.

As mentioned in the Introduction Section, there are at least three phases confirmed in this temperature range: *Pnma*, *I*4/*mcm* and *Pm*$\overline{3}$*m*. To analyse this and the role of MA in the transitions, several ND patterns were sequentially collected during the warming process from 2 to 250 K with a temperature interval of ≈ 4.5 K. A first analysis of all patterns was made from the 2D plots shown in Fig. 5, where the phase transitions are observed. The tetragonal to cubic transition occurs abruptly between the patterns collected at 228.5 and 233.1 K. This temperature transition is in agreement with previous results from DSC, Vibrational Spectroscopy and crystallographic studies^19,24,27,37^. Otherwise, the transition from orthorhombic to tetragonal is diffuse and it is spread in a wide temperature range (from 146.6 to 155.6 K); moreover, the pattern collected at 151.1 K shows an intermediate situation but it was not possible to index it. This is highlighted in Fig. 5 for different reflections. This situation is similar to that found by us from synchrotron x-ray diffraction data at 150 K^21^.

**Figure 6:**
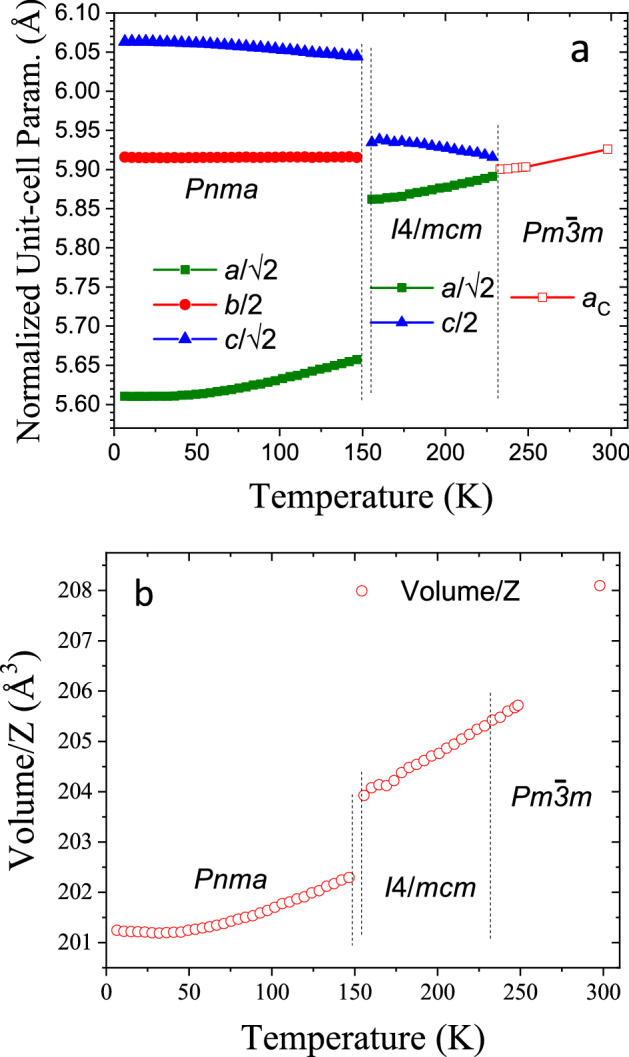
(**a**) Thermal evolution of *a*, *b* and *c* unit-cell parameters and (**b**) unit-cell volume of the orthorhombic phase.

The remaining patterns were correctly fitted with the previously reported space groups. The results are plotted in Fig. 6, where the thermal evolution of normalized unit-cell parameters and volume/Z are illustrated. Up to 146.6 K, the patterns were successfully fitted within an orthorhombic symmetry in the *Pnma* space group, based on the model presented in Table 2 and with no need of establishing any rigid body’s constrains. In this symmetry, the three unit-cell parameters remain in a plateau up to 40 K; then, while *a* parameter increases, the *c* axis undergoes a subtle reduction; conversely, the *b* parameter remains virtually constant. See Fig. 6a.

This particular thermal evolution of the unit-cell parameters is certainly unexpected; however, it can be well related to the MA position and its interactions with the inorganic framework. For instance, as shown in Fig. 4, the MA unit is lying on the *a*-*c* plane and it can be only shifted within this plane; hence, it is not surprising that the *b* parameter hardly changes in this temperature range. On the other hand, the opposite evolution of *a* and *c* parameters are due to the movement of MA in this plane; as temperature decreases the MA alignment comes near to the *c* axis with the consequent increase of *c* parameter and, simultaneously, the decrease of *a* parameter. This effect also can be observed in the octahedral tilts, as illustrated in Fig. S2, where the Pb–Br–Pb angles are shown. It is possible to observe that the Pb–Br2–Pb angle presents greater changes than Pb–Br1–Pb; this fact is also in agreement with the unit-cell parameters evolution.

The unit-cell volume evolution (Fig. 6b) remains constant up to 40 K and then this increase is the result of as a compromise between the unit-cell parameters behaviour.

**Figure 7:**
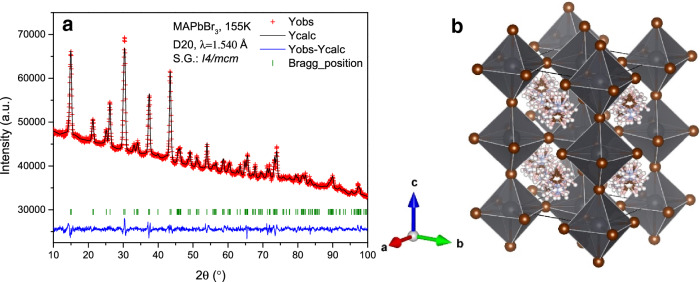
(**a**) Observed (crosses) calculated (black line) and difference (blue line) profiles after the Rietveld refinement from NPD data at 155.6 K, corresponding to the tetragonal (*I4/mcm*) symmetry. (**b**) View of the tetragonal unit-cell of the corresponding crystallographic model.

**Figure 8:**
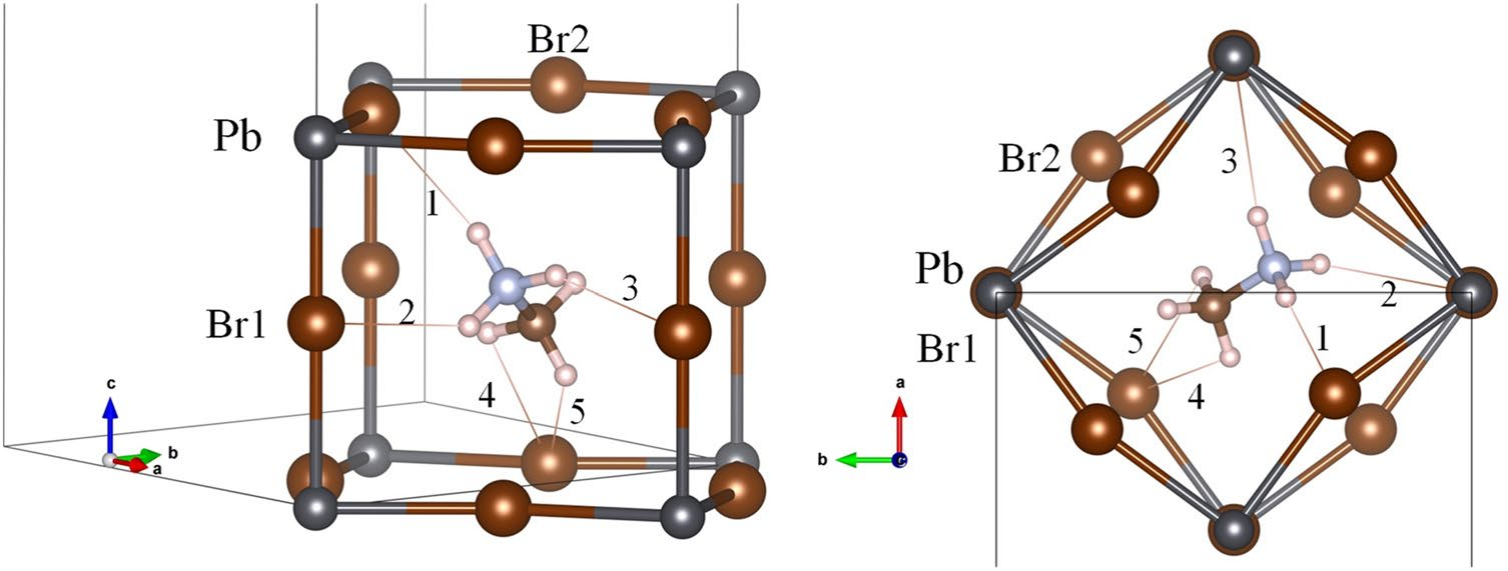
Two views of the tetragonal crystal structure of MAPbBr_3_, highlighting the H-bond interactions with adjacent Br atoms.

After an abrupt phase transition, which will be analysed later on, the tetragonal phase is observed above 155.6 K, which is described in the *I*4*/mcm* space group. In this case, the MA unit was better modelled using the rigid body formalism, unveiling four possible positions of the organic cation. This refinement strategy was needed due to the delocalization of MA in this tetragonal phase. Thereby, it is possible to obtain a more realistic description of the MA situation within the inorganic framework. This organic unit was refined in both position and direction. As representative of this temperature range, Fig. 7 shows the Rietveld pattern refinement at 155.6 K together with a schematic view of the tetragonal crystal structure. Figure 8 shows a single MA unit in the inorganic framework where the main H-bond interactions are highlighted.

In the *I*4*/mcm* space group, Pb atoms are placed in 4*c* (0, 0, 0), whereas Br1 and Br2 atoms are located at 4*c* (0, 0, 1/4) and 8* h* (x, x + 1/2, 0) Wyckoff sites, respectively. The C, N and H atoms forming the organic unit are in 32* m* (x, y, z). The transition to the *I*4*/mcm* space group involves an in-phase octahedral tilt along the *c*-axis, typified as a^0^a^0^c^+^ in Glazer’s notation^38^. The complete crystallographic parameters are included in Table 5**.**

**Table 5:** Crystallographic data for MAPbBr_3_ phase in the tetragonal system (*I*4*/mcm)* from NPD at 155.6 K.

	*x*	*y*	*z*	*U*_*iso*_	*f*_*occ*_
Pb1	0	0	0	0.0053 (6)	1
Br1	0	0	0.25	0.0584 (19)	1
Br2	0.28909 (6)	0.78910 (6)	0	0.059 (4)	1
C1	− 0.0401	0.53029	0.21642	0.001 (4)	0.125
N1	0.08945	0.45138	0.28560	0.001 (4)	0.125
H1	0.11534	0.52114	0.35206	0.101 (5)	0.125
H2	− 0.0660	0.46053	0.14997	0.101 (5)	0.125
H3	− 0.1391	0.54472	0.26369	0.101 (5)	0.125
H4	− 0.0013	0.63802	0.18966	0.101 (5)	0.125
H5	0.18843	0.43695	0.23833	0.101 (5)	0.125
H6	0.05064	0.34365	0.31237	0.101 (5)	0.125

	U^11^	U^22^	U^33^	U^12^	
**Atomic displacement parameters (Å**^**2**^**)**					
Pb	0.0076 (9)	0.0076 (9)	0.00071	0	
Br1	0.087 (3)	0.087( 3)	0.00071	0	
Br2	0.036 (3)	0.036 (3)	0.104(7)	0.024 (3)	
R_p_ = 0.657%, R_wp_ = 0.891%, χ^2^ = 3.41, R_Bragg_ = 4.38%					

*a* = 8.2815(5) Å, *c* = 11.8508(8) Å and V = 812.77(8) Å^3^.

This tetragonal phase is retained from 155.6 up to 228.5 K, where a sequential refinement reveals the evolution of MAPbBr_3_ with temperature. Figure 6 shows the variation of the unit-cell parameters, *a* and *c*, and the unit-cell volume. The *a* parameter increases with temperature, while the *c* parameter slightly decreases. As a consequence, the unit-cell volume evolution increases with temperature.

Finally, the NPD patterns collected from 233.1 to 250.0 K exhibit a cubic crystal structure. Figure 6 shows the unit-cell parameter and volume variation. All parameters regularly increase upon warming up, as is expected by the thermal expansion.

In addition, this sequential analysis also reveals some aspects of these phase transitions. While the T-C transition is smooth, the O-T one is abrupt, displaying a break in the unit-cell parameter and volume cell evolution, see Fig. 6. This behaviour is known and can be related to the change from fixed to delocalized MA cation. However, as mentioned in the Introduction section, several crystallographic aspects of this transition remain unknown. In our previous report on the structural evolution of MAPbBr_3_ from high-angular resolution SXRD, the pattern collected at 150 K was not compatible with the *Pnma* or *I*4/*mcm* space groups^21^. This was also observed in our sequentially acquired NPD data, at 151.5 K, as highlighted in Fig. 5. This fact prompted a more detailed inspection in this narrow temperature range, in order to unveil certain features regarding this transition.

### Sequential analysis from 145 to 157 K (ΔT ≈ 0.5 K)

**Figure 9:**
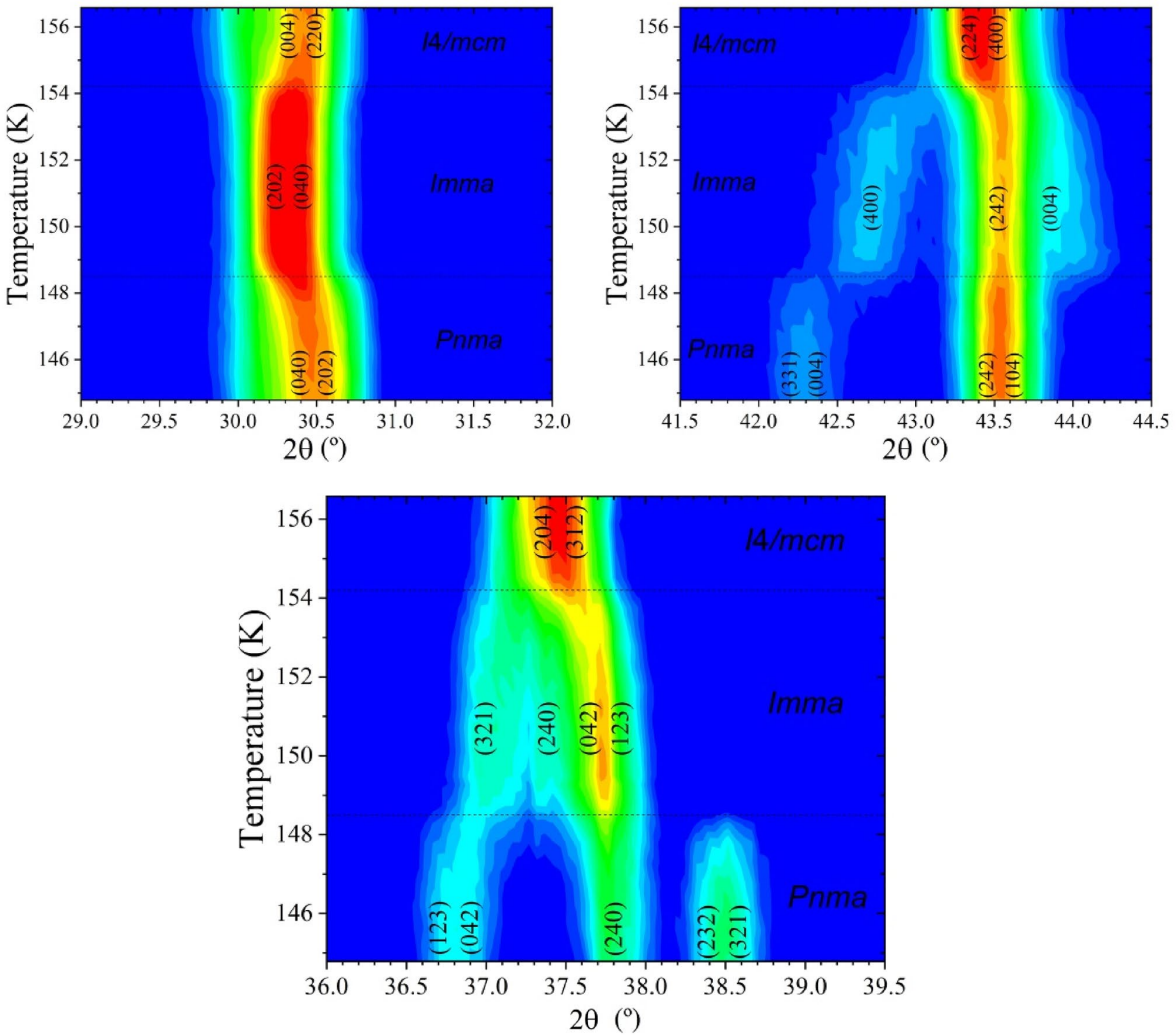
2D maps showing the evolution of three selected NPD angular regions within the 135–156 K temperature range, highlighting the *Pnma-Imma-P4/mmm* phase transitions for MAPbBr_3_.

An additional sequential analysis was performed with a shorter temperature interval (≈ 0.5 K) around the orthorhombic/tetragonal transition. Figure 9 displays the 2D plot of the same three angular ranges shown in Fig. 5, exhibiting the evolution of several diffraction peaks within this temperature region. These additional measurements unveil the existence of an additional crystallographic phase in the 148.5–155.2 K temperature range.

Several works report on a possible coexistence of the orthorhombic and tetragonal phases in this interval^22,26^; we therefore considered the cited possibility. However, it was not possible to accomplish an accurate refinement using this two-phase approach. In other cases, different structural proposals (orthorhombic or tetragonal)^23–25^ described for this short temperature range did not allow fitting the ND patterns.

On the other hand, the more recent analyses over this issue were made for Gou and Wiedemann et al*.* who proposed an incommensurate structure^27,28^. This last work reports an incommensurately modulated structure in the (3 + 1)D superspace group *Imma*(00γ)s00 at 150 K from single crystal X ray diffraction data^28^.

Based on this report, we managed to fit the patterns with a derived commensurate *Imma* model, reaching a satisfactory result. A careful inspection of the patterns showed no evidence of an incommensurate structure. Indeed, no additional peaks of a modulated phase are observed in our data set. Thus, we concluded that our perovskite, according to NPD data, exhibits a low-temperature phase (2–148.3 K) defined in a *Pnma* orthorhombic symmetry, a novel orthorhombic *Imma* phase (148.8–153.8 K), a tetragonal structure defined in the *I*4*/mcm* space group (154.5–228.5 K) and a high-temperature phase (233.1–300 K) in a cubic *Pm*$\overline{3}$*m* symmetry. The evolution of some selected reflections across the three space groups is displayed in the 2D plots of Fig. 9, illustrating the complexity of the MAPbBr_3_ crystallographic behaviour in this narrow temperature region.

Within this framework, we assembled a *Imma* model based upon the reported orthorhombic structure^28^. In this space group, Pb atoms are placed in 4*a* (0, 0, 0), whereas Br1 and Br2 atoms are located at 4*e* (0, 1/4, z) and 8* g* (1/4, y, 1/4) Wyckoff sites, respectively. The MA group is delocalized around (0, 1/4, 1/5) position. The transition to the *Imma* space group involves an in-phase octahedral tilt along *c*-axis, typified as a^0^b^−^c^−^ in Glazer’s notation^38^.

**Figure 10:**
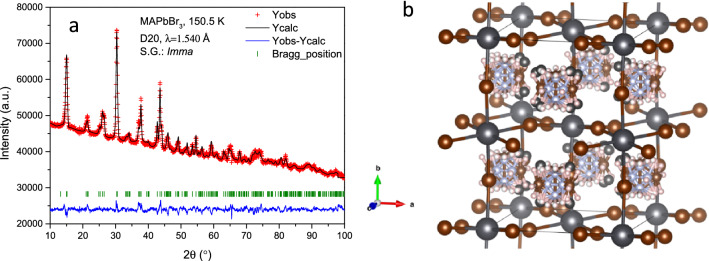
(**a**) Observed (crosses) calculated (black line) and difference (blue line) profiles after the Rietveld refinement from NPD data at 150.5 K, corresponding to orthorhombic (*Imma*) symmetry. (**b**) View of the corresponding crystallographic model.

Additionally, by means of Difference Fourier Maps (DFM) from NPD data, we were able to discern the organic cation, revealing the existence of two distinct delocalized MA groups, in two different positions. The elucidated organic units were refined using the rigid body approach, giving satisfactory results; see Fig. 10a for a Rietveld refinement at 150.5 K and Fig. 10b for the illustrated crystallographic model. The corresponding crystallographic information is listed in Table S1.

**Figure 11:**
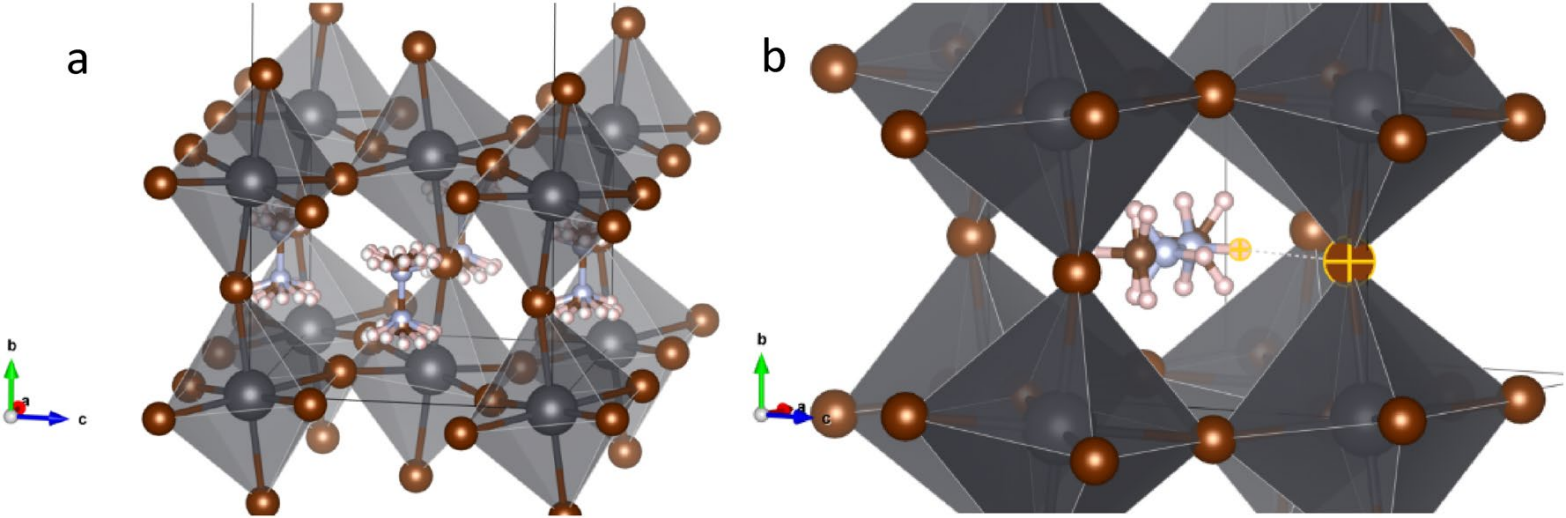
Views of the *Imma* crystallographic model with the two possible configurations for the MA organic units: (**a**) delocalized along the *b* axis and (**b**) delocalized along the *a* axis.

Thus, from a trial and error analysis of the MA position and subsequent DFM calculations, it was possible to find two MA units with different delocalization. As shown in Fig. 11a, one of the MA units is twofold delocalized along the *b*-axis, with the H atoms directed towards each of the four Br atoms. The other MA molecule is also twofold delocalized along the *a*-axis, with a small tilt of about 10°, see Fig. 11b. This MA tilt explains the possible formation of H-bonds and the distortion of the PbBr_6_ structure. The shortest Br···H distance is highlighted in Fig. 11b, showing the correlation between the MA position and the inorganic PbBr_3_ distortion.

**Figure 12:**
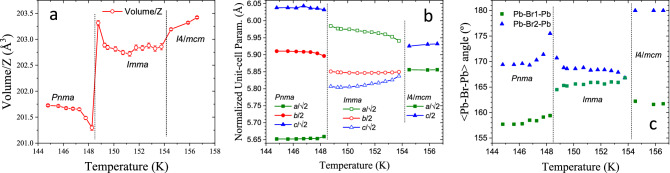
Thermal evolution in the 144–156.5 K range, comprising the *Pnma-Imma* and *Imma-I*4/*mcm* phase transitions of (**a**) the unit-cell volume/Z, (**b**) normalized unit-cell parameters and (**c**) the Pb–Br1–Pb and Pb–Br2–Pb angles.

Figure 12 shows three plots that compare structural details of the low-temperature crystallographic phases, illustrating the behaviour of the novel orthorhombic *Imma* phase, compared to the already known *Pnma* and *I/*4*mcm* phases. The thermal evolution of these parameters completes that previously shown in Figs. 6 and S2. Figure 12a displays the variation of the mean unit-cell volume/Z; there is a general expansion as temperature increases, with some fluctuations around the phase transition due to the rearrangement of the unit cell. Figure 12b illustrates the dependence of the unit-cell parameters with temperature; they are displayed in a pseudocubic form for a better comparison. Within the narrow temperature region for this intermediate *Imma* phase, the *a* parameter decreases with temperature whilst *b* and *c* increase. Figure 12c shows the evolution of the Pb–Br1–Pb and Pb–Br2–Pb angles.

Despite the fact that the *Imma* structure seems to be disruptive in the frequent space groups sequence observed in perovskite compounds, its symmetry presents similarities with both *Pnma* and *I*4/*mcm* space groups. This fact can be observed in Fig. 12 and mainly in the unit-cell parameters evolution (Fig. 12a). Also, is possible observe that this intermediate symmetry can be understood as an orthorhombic phase that retains or stabilizes the MA delocalization. Hence, in terms of MA, this phase is the lowest temperature delocalized state before reaching a fixed position in *Pnma* phase. This phase transition corresponds to the strongest peak observed in the DSC curve (Fig. 1), which was assigned to order–disorder of the MA units. An illustrative scheme of the MA delocalization sequence is illustrated in Figure S3, which surely induces the transition pathway in the inorganic framework.

### H-bond thermal evolution

**Figure 13:**
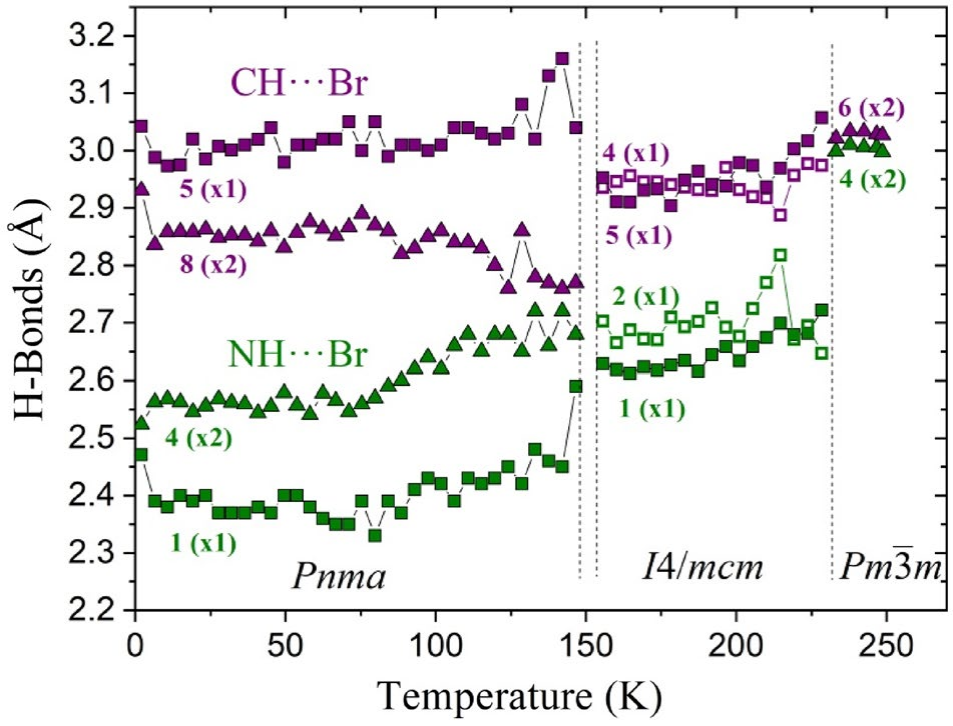
Thermal evolution of H-bond distances in the 2–250 K temperature range. The NH···Br and NH···Br bonds are plotted in green and purple colours, respectively. The single and twofold H-bond interactions are plotted with squares and triangles, respectively.

It is well known that the H-bond interactions in hybrid perovskite materials play a paramount role in the stability of the crystal structures and their phase transitions. Hence, considering the H-bond interactions above described (2 K, 155.6 and 300 K), we now analyse the thermal evolution of these parameters in the 2–250 K temperature range. Figure 13 shows the H···Br as a function of temperature. The label numbers in this Figure match those used in Figs. 2, 4 and 8 for RT, 155.5 and 2 K, respectively, and the number in parenthesis indicates if the H-bonds are single or twofold. It is possible to observe a progressive splitting in the NH···Br and CH···Br distances. While at high temperature there are not substantial differences when the H is bonded to a nitrogen or a carbon, at lower temperatures this difference takes relevance. This difference is moderate in the tetragonal symmetry, but it increases in the orthorhombic phase. This behaviour joins two aspects of the crystal structure at lower temperatures. First, the MA delocalization, presented in the cubic and tetragonal symmetry, avoids the formation of strong H-bond interactions. Then, in the orthorhombic phase the delocalization disappears, enabling the interaction between the MA unit and the PbBr_6_ lattice. The second aspect is the distortion of the inorganic framework, evidenced in the octahedral tilting or in the Br–Pb–Br angles (Fig. S2). Contrasting both parameters (Figs. S2 and 13) it is possible to confirm that the H-bond formation is enabled by the PbBr_6_ lattice distortion or vice versa. Anyway, this aspect reveals the structural complexity of these technological attractive hybrid perovskites.

## Conclusions

We have synthesized a well-crystallized powder and single crystals of the hybrid MAPbBr_3_ perovskite, and its crystallographic features have been determined from neutron diffraction in the 2–300 K temperature range. The NPD data, sensitive to H positions, allowed us to deepen into the configuration of the MA units within the inorganic cages and to study the H-bond interactions, unveiling strong H···Br links. In particular, the low-temperature *Pnma* orthorhombic phase presents a localized MA molecule in a unique position, refined without any rigid body constraints. In this symmetry, the MA is delocalized along [110] positions, in contrast with the tetragonal and cubic phases. The tetragonal structure was described in the *I*4*/mcm* space group, and the MA units were modelled using the rigid body formalism, unveiling four possible locations. The *Pm*$\overline{3}$*m* cubic structure was also followed with NPD data, and it was extensively studied from single-crystal ND at room temperature. In addition, a novel *Imma* orthorhombic phase was identified in the 148–155 K interval. For the first time, we present the complete crystal resolution of this intermediate *Imma* structure, including a full description of organic cation position and its delocalization. We achieved a satisfactory refinement of the structure, including the situation of the organic molecules within that range, giving a comprehensive overview of the crystal structure evolution and phase transitions of MAPbBr_3_ in the 2–300 K temperature range.

## Supplementary Information

Supplementary Information.

## Data Availability

The datasets generated and/or analysed during the current study are available in the COD repository [Crystallography Open Database: Information card for entry 3000390].
