# Supplementary material for: Crystal structure thermal evolution and novel orthorhombic phase of methylammonium lead bromide, CH3NH3PbBr3
Source: Sci Rep. 2022 Nov 4;12:18647. doi: 10.1038/s41598-022-21544-2 (PMC9636425; doi:10.1038/s41598-022-21544-2)
Supplement: Supplementary file 1 — Supplementary Information. [file 41598_2022_21544_MOESM1_ESM.docx]

**Supplementary Information**

**Crystal structure thermal evolution and novel orthorhombic phase of methylammonium lead bromide, CH_3_NH_3_PbBr_3_**

*Carmen Abia,^1,3^ Carlos A. López,^1,2*^, Laura Canadillas-Delgado^3^, María T. Fernández-Diaz^3^ and José A. Alonso^1^**

*^1^ Instituto de Ciencia de Materiales de Madrid, CSIC, Cantoblanco 28049 Madrid, Spain.*

*^2^ INTEQUI, (UNSL-CONICET) and Facultad de Química, Bioquímica y Farmacia, UNSL, Almirante Brown 1455, 5700, San Luis, Argentina.*

*^3^Institut Laue Langevin. 38042 Grenoble Cedex, France.*

**Figure S1:** Two views of the orthorhombic crystal structure of MAPbBr_3_ at 2 K, showing negative (hydrogens) and positive (C and N atoms) areas in the Difference Fourier Maps, suggesting the MA position along [10-1] (left) and [010] (right) directions.

**Table S1:** Crystallographic data for MAPbBr_3_ phase in the orthorhombic system (*Imma)* from NPD at 150.5 K.

*a* = 8.445(1) Å, *b* = 11.692(1) Å, *c* = 8.223(1) Å and V = 811.0(2) Å^3^

|  | ***x*** | ***y*** | ***z*** | ***U_iso_*** | ***f_occ_*** |
| --- | --- | --- | --- | --- | --- |
| **Pb1** | 0 | 0 | 0 | 0.0013 | 1 |
| **Br1** | 0 | 0.25 | 0.955(2) | 0.060(3) | 1 |
| **Br2** | 0.25 | 0.0248(9) | 0.25 | 0.060(3) | 1 |
| **N1** | 0 | 0.19386 | 0.5 | 0.065(5) | 0.2143 |
| **C1** | 0 | 0.31873 | 0.5 | 0.065(5) | 0.2143 |
| **H1** | 0.07661 | 0.16631 | 0.42123 | 0.101 | 0.2143 |
| **H2** | −0.0766 | 0.34629 | 0.57877 | 0.101 | 0.2143 |
| **H3** | 0.02804 | 0.16631 | 0.60760 | 0.101 | 0.2143 |
| **H4** | −0.1047 | 0.16631 | 0.47117 | 0.101 | 0.2143 |
| **H5** | 0.10465 | 0.34629 | 0.52883 | 0.101 | 0.2143 |
| **H6** | −0.0280 | 0.34629 | 0.39240 | 0.101 | 0.2143 |
| **N1** | −0.0562 | 0.25000 | 0.50000 | 0.065(5) | 0.7857 |
| **C1** | 0.11410 | 0.25000 | 0.46949 | 0.065(5) | 0.7857 |
| **H1** | −0.0752 | 0.25000 | 0.61648 | 0.101 | 0.7857 |
| **H2** | 0.13310 | 0.25000 | 0.35302 | 0.101 | 0.7857 |
| **H3** | −0.1031 | 0.31777 | 0.45186 | 0.101 | 0.7857 |
| **H4** | 0.16099 | 0.18223 | 0.51763 | 0.101 | 0.7857 |
| R_p_ = 0.85%, R_wp_ = 1.14%, χ^2^ = 5.57, R_Bragg_ = 8.09% | | | | | |

**Figure S2**: Thermal evolution of Pb–Br–Pb angles.


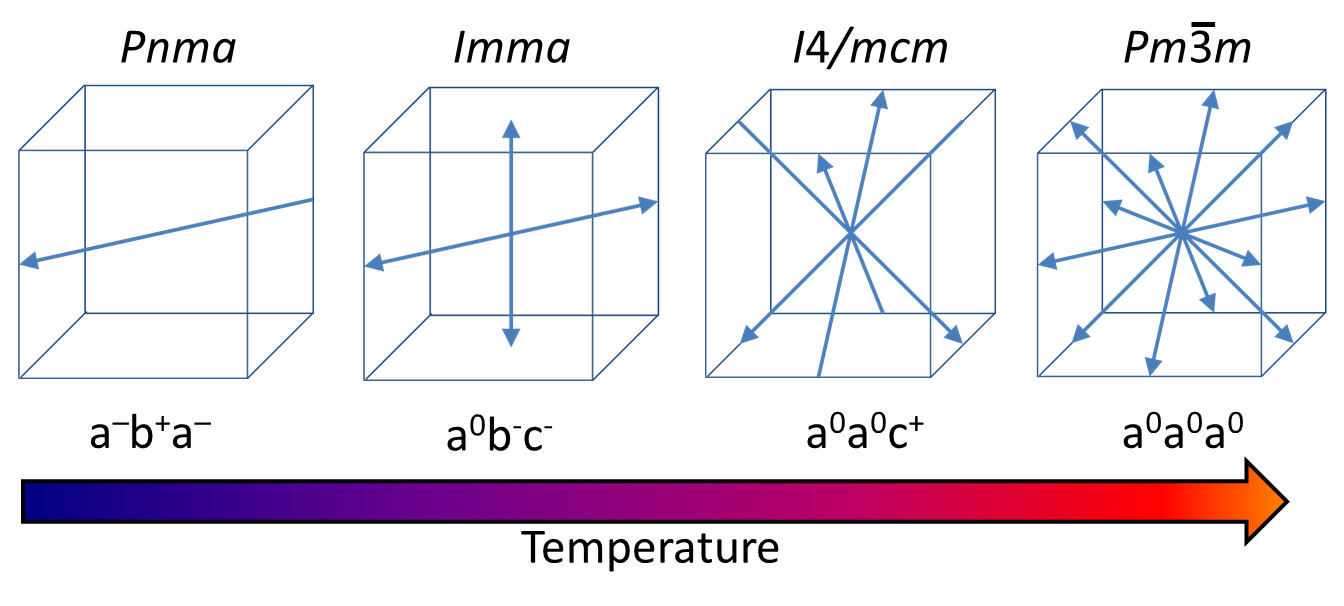


**Figure S3:** Schematic representation of MA delocalization in the different space groups along the sequence of transition observed in MAPbBr_3_.
